# Assessment of Clinician Diagnostic Concordance With Video Telemedicine in the Integrated Multispecialty Practice at Mayo Clinic During the Beginning of COVID-19 Pandemic From March to June 2020

**DOI:** 10.1001/jamanetworkopen.2022.29958

**Published:** 2022-09-02

**Authors:** Bart M. Demaerschalk, Andrew Pines, Richard Butterfield, Jack M. Haglin, Tufia C. Haddad, James Yiannias, Christopher E. Colby, Sarvam P. TerKonda, Steve R. Ommen, Matthew S. Bushman, Troy G. Lokken, Rebecca N. Blegen, Mekenzie D. Hoff, Jordan D. Coffey, Greg S. Anthony, Nan Zhang

**Affiliations:** 1 Department of Neurology and Center for Digital Health, Mayo Clinic College of Medicine and Science, Phoenix, Arizona; 2 Mayo Clinic Alix School of Medicine, Phoenix, Arizona; 3 Now with Department of Psychiatry, Brigham & Women’s Hospital, Boston, Massachusetts; 4 Division of Clinical Trials and Biostatistics, Department of Quantitative Health Sciences, Mayo Clinic College of Medicine and Science, Phoenix, Arizona; 5 Department of Orthopedic Surgery, Mayo Clinic College of Medicine and Science, Phoenix, Arizona; 6 Department of Medical Oncology and Center for Digital Health, Mayo Clinic College of Medicine and Science, Rochester, Minnesota; 7 Department of Dermatology and Center for Digital Health, Mayo Clinic College of Medicine and Science, Phoenix, Arizona; 8 Department of Pediatric and Adolescent Medicine, Neonatology, Critical Care, and Center for Digital Health, Mayo Clinic College of Medicine and Science, Rochester, Minnesota; 9 Department of Surgery and Plastic and Reconstructive Surgery, Center for Digital Health, Mayo Clinic College of Medicine and Science, Jacksonville, Florida; 10 Department of Cardiovascular Medicine and Center for Digital Health, Mayo Clinic College of Medicine and Science, Rochester, Minnesota; 11 Center for Digital Health, Mayo Clinic College of Medicine and Science, Rochester, Minnesota

## Abstract

**Importance:**

There was a shift in patient volume from in-person to video telemedicine visits during the COVID-19 pandemic.

**Objective:**

To determine the concordance of provisional diagnoses established at a video telemedicine visit with diagnoses established at an in-person visit for patients presenting with a new clinical problem.

**Design, Setting, and Participants:**

This is a diagnostic study of patients who underwent a video telemedicine consultation followed by an in-person outpatient visit for the same clinical problem in the same specialty within a 90-day window. The provisional diagnosis made during the video telemedicine visit was compared with the reference standard diagnosis by 2 blinded, independent medical reviewers. A multivariate logistic regression model was used to determine factors significantly related to diagnostic concordance. The study was conducted at a large academic integrated multispecialty health care institution (Mayo Clinic locations in Rochester, Minnesota; Scottsdale and Phoenix, Arizona; and Jacksonville, Florida; and Mayo Clinic Health System locations in Iowa, Wisconsin, and Minnesota) between March 24 and June 24, 2020. Participants included Mayo Clinic patients residing in the US without age restriction. Data analysis was performed from December 2020 to June 2021.

**Exposures:**

New clinical problem assessed via video telemedicine visit to home using Zoom Care Anyplace integrated into Epic.

**Main Outcomes and Measures:**

Concordance of provisional diagnoses established over video telemedicine visits compared against a reference standard diagnosis.

**Results:**

There were 2393 participants in the analysis. The median (IQR) age of patients was 53 (37-64) years; 1381 (57.7%) identified as female, and 1012 (42.3%) identified as male. Overall, the provisional diagnosis established over video telemedicine visit was concordant with the in-person reference standard diagnosis in 2080 of 2393 cases (86.9%; 95% CI, 85.6%-88.3%). Diagnostic concordance by *International Statistical Classification of Diseases and Related Health Problems, Tenth Revision *chapter ranged from 64.7% (95% CI, 42.0%-87.4%) for diseases of the ear and mastoid process to 96.8% (95% CI, 94.7%-98.8%) for neoplasms. Diagnostic concordance by medical specialty ranged from 77.3% (95% CI, 64.9%-89.7%) for otorhinolaryngology to 96.0% (92.1%-99.8%) for psychiatry. Specialty care was found to be significantly more likely than primary care to result in video telemedicine diagnoses concordant with a subsequent in-person visit (odds ratio, 1.69; 95% CI, 1.24-2.30; *P* < .001).

**Conclusions and Relevance:**

This diagnostic study of video telemedicine visits yielded a high degree of diagnostic concordance compared with in-person visits for most new clinical concerns. Some specific clinical circumstances over video telemedicine were associated with a lower diagnostic concordance, and these patients may benefit from timely in-person follow-up.

## Key Points

**Question:**

How concordant to an in-person diagnosis are provisional diagnoses established at a video telemedicine visit for patients presenting with a new clinical problem?

**Findings:**

In this diagnostic study of 2393 patients who underwent a video telemedicine consultation followed by an in-person outpatient visit for the same clinical problem in the same specialty within a 90-day window, the provisional diagnosis established over video telemedicine visit matched the in-person reference standard diagnosis in 86.9% of cases.

**Meaning:**

These findings suggest that video telemedicine visits yield a high degree of diagnostic concordance to in-person visits for most new clinical concerns.

## Teaser

This diagnostic study examines the concordance of provisional diagnoses established at a video telemedicine visit with diagnoses established at an in-person visit for patients presenting with a new clinical problem.

## Introduction

During the first months of the COVID-19 pandemic, in response to the increased risk of viral exposure to patients and clinicians associated with in-person visits and to conserve personal protective equipment, many health care organizations transitioned appropriate in-person patient appointments to video telemedicine visits. At its height in April 2020, telehealth usage was estimated to have increased 20-fold in the US.^1,2,3^ Video visits at Mayo Clinic in particular increased by 10 880%.^4^ This shift of patient volume from in-person clinics to video telemedicine services will likely have a lasting impact on how health care is delivered in the future, but gaps in telemedicine research may limit clinicians’ ability to make evidence-based management plans tailored to digital health care.

One of the principal concerns has been the limited data available regarding the accuracy with which clinicians diagnose patients’ diseases and conditions through video telemedicine visits and regarding the concordance between a video telemedicine diagnosis and that established through an in-person visit.^5,6,7^ Large-scale, multispecialty data on the concordance of video telemedicine diagnoses with in-person visit diagnoses would help clinicians make evidence-based decisions regarding for which patients, in which clinical specialties, and for which types of clinical problems video telemedicine visits are likely to be sufficient for diagnosis.^8,9,10,11,12^ Before the COVID-19 pandemic, studies^13,14,15^ on the accuracy and concordance of video telemedicine diagnosis were limited by sample size, breadth of presenting diseases and clinical problems, representativeness of primary and specialty medical and surgical practices, and clinical practice implementation. A recent comprehensive report^16^ on the state of telehealth identified quantifying the diagnostic accuracy of telehealth as a critical research gap. The increase in telemedicine volume during the COVID-19 pandemic enabled our institution to rapidly gather data on video telemedicine visits on a larger scale than had previously been available.

## Methods

The Mayo Clinic institutional review board (IRB) application was reviewed by expedited review procedures on April 8, 2020, and was determined to be exempt from the requirement for IRB approval and informed consent because the data were deidentified (45 CFR 46.104d, category 4). The Mayo Clinic IRB approved a waiver of Health Insurance Portability and Accountability Act (HIPAA) authorization in accordance with applicable HIPAA regulations. The Standards for Reporting of Diagnostic Accuracy (STARD) 2015 reporting guidelines for reporting diagnostic studies were followed.

### Patient Population

To be eligible for inclusion in the study, Mayo Clinic patients residing in the US required both a video telemedicine to home consultation for a new clinical indication via Mayo Clinic Care Anyplace between March 24, 2020, and June 24, 2020, and an in-person follow-up visit in the same clinical department for the same indication within 90 days of the telemedicine consultation. Patients who had checked a box preferring no research participation at the time of electronic registration to Mayo Clinic were excluded. Mayo Clinic locations were in Rochester, Minnesota; Scottsdale and Phoenix, Arizona; and Jacksonville, Florida; and Mayo Clinic Health System locations were in Iowa, Wisconsin, and Minnesota.

### Modes of Clinical Assessment

Video telemedicine was conducted using standard Zoom Mayo Clinic Care Anyplace integrated into Epic.^17^ Patients were scheduled for their clinician referral or self-referral appointments through Epic and checked in for their appointments through the Mayo Clinic portal.^18^ Patients used desktop computers, laptop computers, tablets, or smartphones in their place of residence, while clinicians used desktop and laptop computers in their offices. No peripheral attachments or devices (eg, stethoscopes, otoscopes, or ophthalmoscopes) were available during the telemedicine assessments. In-person visits were similarly scheduled through Epic and conducted on-site at any of the Mayo Clinic campuses. The description of applicable digital health technology and operations from patient and clinician standpoints has been previously published.^17,18^

### Clinicians and Data Collection

For this study, clinicians included Mayo Clinic–employed consultant physicians (medical doctors and doctors of osteopathy) and advanced practice providers (nurse practitioners and physician assistants). Data from video telemedicine and in-person consultations for each included patient were extracted from the electronic medical record (EMR) Epic and auto-populated into a Research Data Capture (REDCap) database. These data included medical record number, patient demographics, primary diagnosis *International Statistical Classification of Diseases and Related Health Problems, Tenth Revision (ICD-10)* code(s), day and time of the encounter, clinician type and characteristics, including years since certification, years of employment at Mayo Clinic, experience conducting telemedicine visits, and clinical department.

Twenty-seven trained third-year and fourth-year medical students from Mayo Clinic Alix School of Medicine familiar with Epic reviewed cases to abstract additional diagnostic data, including method of reference standard diagnosis, and make a blinded determination of the diagnostic agreement of the provisional telemedicine diagnosis and the reference standard in-person follow-up visit diagnosis. Medical reviewer training included two 1-hour virtual workshops conducted by lead study investigators describing in detail the workflow necessary to review the EMR, identify study-related data and definitions, decision-making, and data extraction. The definition of telemedicine provisional diagnosis was the diagnosis offered by the telemedicine clinician at the conclusion of the visit, taking into account the referral indication, chief concern, any diagnostic records, history acquisition, and video examination. The definition of the reference standard diagnosis was the diagnosis offered by the in-person clinician at the conclusion of the visit, taking into account the in-person history acquisition and physical examination, the results of any diagnostic testing available, and any information available in the EMR to the date of in-person visit. In instances where the reviewer determined that the provisional diagnosis was discordant with the reference standard diagnosis, the reviewer further assessed whether the diagnostic discordance carried the potential for morbidity or mortality and whether there was any actual morbidity or mortality. The medical reviewers reviewed the EMR documentation to determine how the clinicians tested their principal provisional diagnosis and probed their provisional differential diagnoses.

In instances when a clinician established a diagnosis without reliance on a physical examination or any diagnostic testing, the modality of diagnosis was listed as “clinician opinion only.” In instances when clinicians documented specific positive physical examination findings consistent with or pathognomonic of a diagnosis, reviewers marked the modality of diagnosis as “physical examination.” In instances when clinicians documented in EMR the use of laboratory testing, diagnostic imaging, cardiac testing (ie, electrocardiogram, Holter monitor, echocardiogram, or cardiac stress test), neurological testing (ie, electroencephalogram or electromyography-nerve conduction study), or pathology (ie, biopsy), reviewers marked these accordingly. Other than the category of “clinician opinion only,” multiple other modalities of diagnosis could be listed and counted in the inventory.

Each case was independently reviewed by 2 blinded medical students. In instances where the 2 reviewers’ assessments of the diagnostic concordance disagreed, the principal investigator reviewed the case in consultation with a specialist from the applicable clinical area. Medical reviewers also had an opportunity to flag any complex cases for tertiary review by the primary investigator. Finally, the primary investigator was assigned to review a random selection of 20% of cases in which the 2 medical student reviewers agreed and did not flag the case. In all applicable cases, the independent determination of the primary investigator was used as the determination of record.

### Statistical Analysis

Patients’ demographics and clinical characteristics along with clinicians’ characteristics were summarized in frequency (percentage), mean (SD), or median (IQR) accordingly. Prevalence-adjusted, bias-adjusted κ coefficients^19^ were calculated between the 2 medical student reviewers and also between the medical student reviewers and the physician reviewer to show interrater agreement. Telemedicine diagnostic concordance and the corresponding 95% CI by patients’ characteristics (ie, adult vs pediatric, sex, and residence) as well as by clinicians’ characteristics (ie, clinician training, setting, specialty, clinical area, and disease area) were estimated using binomial method. The χ^2^ test was used to test whether the telemedicine diagnostic concordances differed by different clinical practices. Logistic regression models with correlated data (patients’ data clustered within clinician) were used to determine patient and clinician characteristics that affected telemedicine diagnostic concordance. Generalized estimating equations method using exchangeable working correlation (to take the within-clinician correlation into account) was used to estimate the model parameters. Patient age, sex, clinician site, clinician type (nonphysician vs physician), case type (surgical vs nonsurgical), patient type (pediatric vs adult), specialty (specialty care vs primary care), telemedicine visit duration, clinicians’ years certified, and clinicians’ prior telemedicine experience were put in the univariate model, and the significant factors from the univariate model were verified in a multivariable model. All statistical analyses were performed using SAS statistical software version 9.4 (SAS Institute). All the hypothesis testing were 2-tailed testing, and *P* < .05 was chosen as the significance level. All statistical analyses were prespecified in the study protocol. Data analysis was performed from December 2020 to June 2021.

## Results

### Case Selection

**Figure 1.**
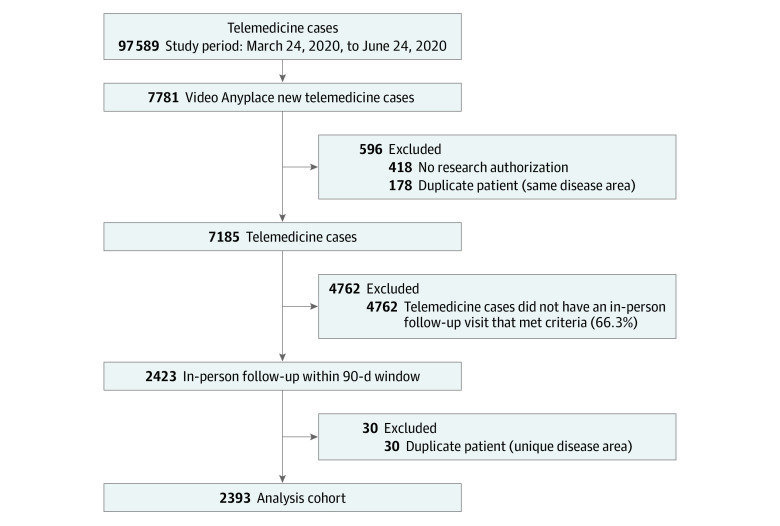
Data Flowchart

Table 1 describes the demographics and characteristics of patients, clinicians, and clinical settings. A total of 97 589 video telemedicine to home consultations took place at our institution between March 24, 2020, and June 24, 2020. Of these, 7781 telemedicine to home consultations were designated for a new health concern. Of these 7781 patients, 418 were excluded because their Mayo Clinic registration records indicated “no research permitted” and 178 were excluded because of duplicate cases, leaving 7185. Of the 7185 patients, 2423 had an in-person follow-up visit within the prespecified 90-day window in the same department for the same health concern. An additional 30 duplicate cases were excluded, leaving 2393 eligible cases in the final cohort. The median (IQR) age of patients was 53 (37-64) years; 1381 (57.7%) identified as female and 1012 (42.3%) identified as male (Figure 1).

**Table 1.** Patient, Clinician, and Case Characteristics

Characteristics	Total, No. (%)
Patients (n = 2393)	
Age at telehealth visit, median (IQR), y	53 (37-64)
Care setting	
Adult	2356 (98.5)
Pediatric	37 (1.5)
Gender	
Male	1012 (42.3)
Female	1381 (57.7)
Clinicians (n = 927)	
Gender	
Male	495 (56.6)
Female	380 (43.4)
Age, mean (SD), y	46.1 (10.6)
Years certified, median (IQR)	13 (7-22)
Length of service at Mayo Clinic, median (IQR), y	9 (4-19)
Clinician type	
Nonphysician	200 (21.6)
Physician	727 (78.4)
Any prior telemedicine experience	116 (12.5)
Included study cases, mean (SD), No.	2.6 (3.0)
Telemedicine visits during study period, median (IQR), No.	27 (12-49)
Clinician concordance, mean (SD), %	87.1 (27.9)
Cases (n = 2393)	
Clinician type	
Nonphysician	408 (17.0)
Physician	1985 (83.0)
Clinician specialty	
Nonsurgical	1689 (70.6)
Surgical	704 (29.4)
Clinician setting	
Primary care	498 (20.8)
Specialist	1895 (79.2)
Telemedicine consultation duration, median (IQR), min	40 (30-60)
Interval between diagnoses, median (IQR), d	28 (13-49)
Modality of diagnosis	
Clinician opinion only	355 (14.8)
Physical examination	1031 (43.1)
Laboratory tests	696 (29.1)
Imaging	957 (40.0)
Cardiac test	172 (7.2)
Neurology test	94 (3.9)
Pathology	212 (8.9)

The eFigure in Supplement 1 depicts the study patients’ home residences by zip code during their respective video telemedicine visits. National representation is evident, with expected clustering in the Midwest, Southwest, and Southeast in regions represented by Mayo Clinic campuses.

### Diagnostic Concordance

The overall concordance of diagnoses between video telemedicine visits and in-person visits was determined to be 86.9% (95% CI, 85.6%-88.3%) (2080 of 2393 visits). Patients with concordant and discordant diagnoses did not differ significantly in terms of sex, Mayo Clinic care site, or location of residence (Table 2).

**Table 2.** Concordance Estimates

Group	Accurate cases, No./total cases, No.	Concordance, % (Wald 95% CI)	*P* value
Patient care setting			
Adult	2051/2356	87.1 (85.7-88.4)	.12
Pediatric	29/37	78.4 (65.1-91.6)	
Patient gender			
Female	1204/1381	87.2 (85.4-88.9)	.66
Male	876/1012	86.6 (84.5-88.7)	
Clinician training			
Nonphysician	357/408	87.5 (84.3-90.7)	.70
Physician	1723/1985	86.8 (85.3-88.3)	
Clinician setting			
Primary care	405/498	81.3 (77.9-84.7)	<.001
Specialist	1675/1895	88.4 (86.9-89.8)	
Clinician specialty			
Nonsurgical	1449/1689	85.8 (84.1-87.5)	.01
Surgical	631/704	89.6 (87.4-91.9)	

In univariate analysis of diagnostic concordance, there were significant differences in concordance between cases seen in specialty care (1675 of 1895 cases [88.4%]) and primary care (405 of 498 cases [81.3%]) (odds ratio [OR], 1.68; 95% CI, 1.27-2.21; *P* < .001) as well as between surgical (631 of 704 cases [89.6%]) and nonsurgical (1449 of 1689 cases [85.8%]) practice settings (OR, 1.38; 95% CI, 1.02-1.87; *P* = .04). After adjustment, there remained a significant difference in concordance between cases seen in specialty care and primary care (accuracy, 88.4% vs 81.3%; OR, 1.69; 95% CI, 1.24-2.30; *P* < .001) but not between surgical and nonsurgical practice settings (accuracy, 89.6% vs 85.8%; OR, 1.22; 95% CI, 0.87-1.71, *P* = .24). The patient’s age was shown to be negatively associated with diagnostic concordance. For every 10-year increase in the patients’ age, the odds of receiving a concordant diagnosis by video telemedicine decreased by 9% (OR, 0.91; 95% CI, 0.85-0.97; *P* = .007). Diagnostic concordance did not significantly vary between clinician types (physicians vs advanced practice providers), adult and pediatric patients, durations of the consultation, or prior video telemedicine experience of the clinician (Table 2). eTable 1 in Supplement 1 presents full logistic regression models results.

The time since clinician certification was categorized using quantiles from the data. Diagnostic concordance was 86.6% (406 of 469 cases) when the clinician had been certified within the past 7 years. The diagnostic concordance was 88.4% (418 of 473 cases) for certification between 7 and 14 years before this study and was 88.6% (507 of 572 cases) for certification between 14 and 23 years. Diagnostic concordance was 84.1% (427 of 508 cases) for certification 23 years or more before this study. Differences between groups were not significant.

Methods of establishing diagnoses at the in-person follow-up visit are presented in Table 1. When an in-person reference standard diagnosis could be satisfactorily established by clinician opinion only, there was a significant increase in the diagnostic concordance between video telemedicine and in-person visits. There was a significant decrease in diagnostic concordance when the method of establishing the reference standard diagnosis necessitated confirmatory pathology, a physical examination, or neurological testing.

**Figure 2.**
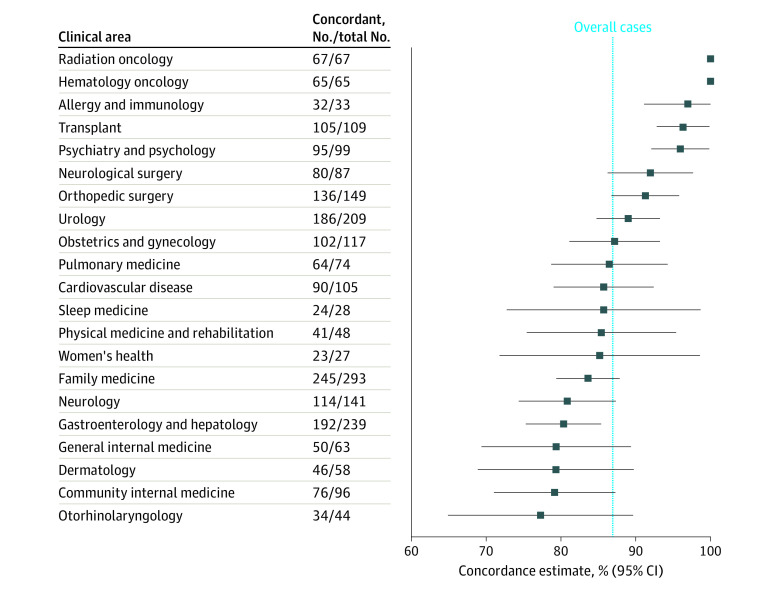
Video Telemedicine Diagnostic Concordance by Clinical Area

For medical specialties with more than 10 cases in the study, diagnostic concordance ranged from 77.3% (95% CI, 64.9%-89.7%) for otorhinolaryngology to 96.0% (95% CI, 92.1%-99.8%) for psychiatry and psychology. The diagnostic concordance by medical specialty is presented in Figure 2 and eTable 2 in Supplement 1.

**Figure 3.**
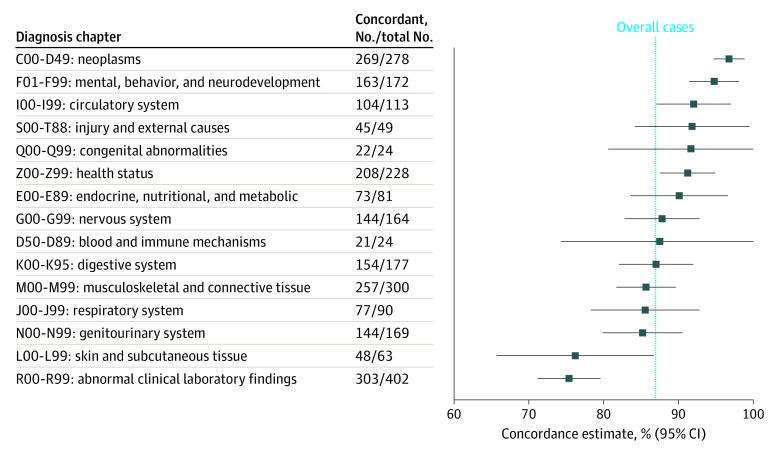
Video Telemedicine Diagnostic Concordance by *International Statistical Classification of Diseases and Related Health Problems, Tenth Revision *Diagnosis Chapter

For *ICD-10* code chapters with more than 10 cases in the study, diagnostic concordance ranged from 64.7% (95% CI, 42.0%-87.4%) for diseases of the ear and mastoid process to 96.8% (95% CI, 94.7%-98.8%) for neoplasms. The diagnostic concordance by *ICD-10* chapter code are presented in Figure 3 and eTable 3 in Supplement 1. A list of specific clinical conditions or diseases with encounter frequency more than 5 patient-cases, and the associated diagnostic concordance is included in eTable 4 in Supplement 1.

### Reviewer Agreement

Adjusted κ agreement between medical student reviewers was strong (0.81). The adjusted κ was moderate in cases where a determination was made by the principal investigator (0.60) and a medical student reviewer (0.64) in instances of discordance between the 2 initial medical reviews. Adjusted κ was strong (0.79) between medical student reviewers and principal investigator in the 20% of cases randomly selected for oversight.

### Morbidity and Mortality

Of all 2393 cases, 313 (13.1%) had video telemedicine provisional diagnoses that were not sufficiently concordant with the reference standard diagnosis established at the in-person visit. Among these cases, 166 of 313 (53.0%) were determined to have had the potential for morbidity and 36 of 166 (21.7%) had actual morbidity. Of 313 cases, 30 (9.6%) were determined to have had the potential for mortality and 3 of 30 (10.0%) had actual mortality during the 90-day interval.

By 6 months after the start of data collection, 31 patients in the cohort had died. For 6 of 31 of these patients, the in-person reference standard diagnosis was not concordant with the provisional video telemedicine diagnosis. Reviewers determined that for 5 of 6 of these patients, the cause of death was unrelated to the video telemedicine consultation or the cause of death could not have been prevented if the patient had instead been seen for an in-person consultation.

## Discussion

In this diagnostic study of a large data set of patients who underwent video telemedicine to home for new clinical problems or presentations at our institution during the COVID-19 pandemic, we found that there was concordance between the provisional diagnosis established during the video telemedicine visit and the diagnosis at the conclusion of a subsequent in-person follow up within 90 days. In 86.9% of cases in our study, the provisional video telemedicine diagnoses matched the reference standard in-person diagnoses. This percentage agreement is consistent with what has been reported in smaller studies that studied diagnostic accuracy of virtual visits in general medical practice.^14^ New patient cases presenting to primary care via telemedicine had a significantly lower diagnostic concordance between telemedicine and in-person visits than did those patient cases presenting first by video telemedicine to specialty clinics. The diagnostic concordance varied between medical specialties and *ICD-10* codes, with oncological, transplant, and psychiatric issues having the most diagnostic concordance between video and in-person visits and otological and dermatological issues having the least diagnostic concordance.

The higher diagnostic concordance between video and in-person visits associated with types of clinical problems or types of diagnoses appears to align with how each diagnosis is confirmed. In diagnoses confirmed through clinician opinion, such as many psychiatric diagnoses, there was a significantly greater concordance between video telemedicine diagnosis and in-person diagnosis. In diagnoses necessitating confirmation through traditional physical examination, neurological testing, and pathology—such as many otological and dermatological diagnoses—there was a significantly decreased concordance between video telemedicine and in-person diagnoses.

Ohta et al^15^ reported that telemedicine can provide the same or similar level of diagnostic concordance as face-to-face practice, 0.75 for telemedicine vs 0.81 for face-to-face. Telemedicine diagnostic concordance was reported in the published literature to be 0.40 for rheumatological complaints,^20^ 0.61 to 0.80 for musculoskeletal complaints,^21^ 0.75 for dermatological complaints,^22^ 0.88 for neurosurgical complaints,^23^ and 0.80 to 1.00 for psychiatric complaints,^24^ consistent with a trend to lower diagnostic concordance for video telemedicine visits compared with in-person visits in specialties requiring a hands-on physical examination or inspection and higher for those without. Our study highlights lower diagnostic concordance for the specialty of otolaryngology, although the 95% CIs around the point estimate were wide. This result is not surprising given the limitations of the video telemedicine to home technology without any peripheral attachments or devices (eg, video-laryngoscopy, video-otoscopy, or video-nasopharyngolaryngoscopy). Ning et al,^25^ however, concluded that studies assessing quality and diagnostic concordance in otolaryngology reported adequate results with significant heterogeneity.

One of the most salient findings in our study was the discrepancy between video telemedicine diagnostic concordance with in-person visits in specialty care (higher concordance) and primary care (lower concordance) clinical settings. This finding was further emphasized by our individual analyses of cases that resulted in morbidity and mortality. There were some cases identified in our primary-care telemedicine program that resulted in morbidity and mortality that might have been mitigated by an initial in-person visit, an observation that was not mirrored in specialty practices.

Research like this study in the current digital health era is reminiscent of historical clinical studies that examined the relative contributions of history taking, physical examination, and laboratory investigation to diagnose new patients presenting to a medical outpatient clinic. Hampton et al^26^ reported that in approximately 82% of new cases, the medical history provided sufficient information to establish an initial diagnosis of a specific disease entity that agreed with the reference standard diagnosis. The physical examination and investigations each proved useful to establish a diagnosis in only 8% and 9% of new cases, respectively.^26^ Peterson et al^27^ reported that in 76% of patients the history led to the final diagnosis, whereas the physical examination and laboratory investigations led to the final diagnosis in 12% and 11% of cases, respectively. Video telemedicine assessments afford ample opportunity for history acquisition but may pose some limits to the comprehensiveness of a traditional in-person physical examination in some specialties.

### Strengths and Limitations

The size of our patient cohort, the breadth of medical and surgical practices, the use of multiple independent blinded medical reviewers, and the individualized methods we used to analyze data allowed for a high degree of confidence in the reported diagnostic concordance of telemedicine video consults. The clinical practice design of this study increases confidence that these results will be generalizable to other practices.

Although the specificity with which we analyzed each case is a strength of this study compared with other large telemedicine studies, further specificity could benefit clinicians by informing on both the categories of diseases that are less accurately diagnosed over telemedicine and common telemedicine pitfalls seen in different specialties. New patients may have been seeking tertiary care at our clinic and may have already had extensive workups at outside facilities. This could limit the generalizability of the results we found in some of the specialty services, such as hematology-oncology, radiation oncology, and transplant medicine.

Another limitation is the potential nonrepresentativeness of the patient group under study. The patients with video telemedicine visits for a new clinical indication for which there was a corresponding in-person visit in the same department for the same indication within 90 days are neither the complete set of video telemedicine visits nor a random sample of those visits. They are instead a potentially nonrepresentative and a nonrandom sample. As a result, the group of patients under study may not be totally representative of all patients undergoing a video telemedicine visit for a new clinical indication. The generalizability of the results should be cautiously interpreted in this context. However, we designed this clinical practice study at the height of the COVID-19 pandemic to be as pragmatic as possible. The specified eligibility criteria were both (1) a video telemedicine visit to home for a new clinical indication and (2) a follow-up visit in person in the same department for the same indication within a 90-day window. The study did exclude patients with a video visit to home who did not return for an in-person visit, who returned for another indication altogether, or who returned to a different department. It is not unreasonable to assume that patients and clinicians most satisfied with the assessment and outcome of a video telemedicine visit for a straightforward and noncomplex clinical issue during COVID-19 may have been less likely to schedule an in-person visit to follow up. We postulate that diagnostic concordance of video telemedicine visits would be higher for patients with straightforward and noncomplex clinical issues.

## Conclusions

In a large database of patients seen for new clinical problems or presentations early during the COVID-19 pandemic, we found 86.9% concordance between provisional diagnoses offered at the time of selected video telemedicine to home assessments and the subsequent in-person reference standard diagnoses determined within 90 days for these visits. These findings suggest that video telemedicine visits to home may be good adjuncts to in-person care. Primary care video telemedicine programs designed to accommodate new patients or new presenting clinical problems may benefit from a lowered threshold for timely in-person direct follow-up in patients suspected to have diseases typically confirmed by physical examination, neurological testing, or pathology.

## Notes

Supplement 1. **eFigure.** Geographic Residence of Patients at Telemedicine Visit **eTable 1. **Logistic Regression Model Results **eTable 2. **Concordance Estimates by Clinical Area **eTable 3. **Concordance Estimates by *ICD* Chapter **eTable 4. **Concordance by *ICD* Code (>10 Cases)

Supplement 2. **Nonauthor Collaborators.** Diagnostic Accuracy of Telemedicine Utilized at Mayo Clinic Alix School of Medicine Study Group Investigators

## References

[1] Patel SY, Mehrotra A, Huskamp HA, Uscher-Pines L, Ganguli I, Barnett ML. Trends in outpatient care delivery and telemedicine during the COVID-19 pandemic in the US. JAMA Intern Med. 2021;181(3):388-391. doi:10.1001/jamainternmed.2020.5928 33196765 PMC7670397

[2] Demeke HB, Merali S, Marks S, . Trends in use of telehealth among health centers during the COVID-19 pandemic—United States, June 26-November 6, 2020. MMWR Morb Mortal Wkly Rep. 2021;70(7):240-244. doi:10.15585/mmwr.mm7007a3 33600385 PMC7891688

[3] Alexander GC, Tajanlangit M, Heyward J, Mansour O, Qato DM, Stafford RS. Use and content of primary care office-based vs telemedicine care visits during the COVID-19 pandemic in the US. JAMA Netw Open. 2020;3(10):e2021476. doi:10.1001/jamanetworkopen.2020.21476 33006622 PMC7532385

[4] Demaerschalk BM, Blegen RN, Ommen SR. Scalability of telemedicine services in a large integrated multispecialty health care system during COVID-19. Telemed J E Health. 2021;27(1):96-98. doi:10.1089/tmj.2020.0290 32795147

[5] Herzer KR, Pronovost PJ. Ensuring quality in the era of virtual care. JAMA. 2021;325(5):429-430. doi:10.1001/jama.2020.24955 33528544

[6] Kahn JM. Virtual visits: confronting the challenges of telemedicine. N Engl J Med. 2015;372(18):1684-1685. doi:10.1056/NEJMp1500533 25923547

[7] Zulman DM, Verghese A. Virtual care, telemedicine visits, and real connection in the era of COVID-19: unforeseen opportunity in the face of adversity. JAMA. 2021;325(5):437-438. doi:10.1001/jama.2020.27304 33528520

[8] Brown EM. The Ontario Telemedicine Network: a case report. Telemed J E Health. 2013;19(5):373-376. doi:10.1089/tmj.2012.0299 23301768

[9] Uscher-Pines L, Mehrotra A. Analysis of Teladoc use seems to indicate expanded access to care for patients without prior connection to a provider. Health Aff (Millwood). 2014;33(2):258-264. doi:10.1377/hlthaff.2013.0989 24493769

[10] Lum HD, Nearing K, Pimentel CB, Levy CR, Hung WW. Anywhere to anywhere: use of telehealth to increase health care access for older, rural veterans. Public Policy Aging Rep. 2020;30(1):12-18. doi:10.1093/ppar/prz030 PMC12447726 40979974

[11] Nord G, Rising KL, Band RA, Carr BG, Hollander JE. On-demand synchronous audio video telemedicine visits are cost effective. Am J Emerg Med. 2019;37(5):890-894. doi:10.1016/j.ajem.2018.08.017 30100333

[12] Harper K, Roof M, Wadhawan N, . Vanderbilt University Medical Center ambulatory teleneurology COVID-19 experience. Telemed J E Health. 2021;27(6):701-705. doi:10.1089/tmj.2020.0382 33216703 PMC8215411

[13] Schoenfeld AJ, Davies JM, Marafino BJ, . Variation in quality of urgent health care provided during commercial virtual visits. JAMA Intern Med. 2016;176(5):635-642. doi:10.1001/jamainternmed.2015.8248 27042813 PMC6842573

[14] Dixon RF, Stahl JE. A randomized trial of virtual visits in a general medicine practice. J Telemed Telecare. 2009;15(3):115-117. doi:10.1258/jtt.2009.003003 19364890

[15] Ohta M, Ohira Y, Uehara T, . How accurate are first visit diagnoses using synchronous video visits with physicians? Telemed J E Health. 2017;23(2):119-129. doi:10.1089/tmj.2015.0245 27351424

[16] Graber M, Schrandt S. Improving telediagnosis: a call to action—final project findings. Society to Improve Diagnosis in Medicine. 2021. Accessed August 2, 2022. https://www.improvediagnosis.org/wp-content/uploads/2021/09/TeleDx-Final-Report-Update.pdf

[17] Lokken TG, Blegen RN, Hoff MD, Demaerschalk BM. Overview for implementation of telemedicine services in a large integrated multispecialty health care system. Telemed J E Health. 2020;26(4):382-387. doi:10.1089/tmj.2019.0079 31433261

[18] Haddad TC, Blegen RN, Prigge JE, . A scalable framework for telehealth: the Mayo Clinic Center for Connected Care response to the COVID-19 pandemic. Telemed Rep. 2021;2(1):78-87. doi:10.1089/tmr.2020.0032 35720756 PMC8989082

[19] Byrt T, Bishop J, Carlin JB. Bias, prevalence and kappa. J Clin Epidemiol. 1993;46(5):423-429. doi:10.1016/0895-4356(93)90018-V 8501467

[20] Graham LE, McGimpsey S, Wright S, . Could a low-cost audio-visual link be useful in rheumatology? J Telemed Telecare. 2000;6(suppl 1):S35-S37. doi:10.1258/1357633001934078 10793966

[21] Russell T, Truter P, Blumke R, Richardson B. The diagnostic accuracy of telerehabilitation for nonarticular lower-limb musculoskeletal disorders. Telemed J E Health. 2010;16(5):585-594. doi:10.1089/tmj.2009.0163 20575726

[22] Oakley AM, Astwood DR, Loane M, Duffill MB, Rademaker M, Wootton R. Diagnostic accuracy of teledermatology: results of a preliminary study in New Zealand. N Z Med J. 1997;110(1038):51-53. 9076285

[23] Wong HT, Poon WS, Jacobs P, . The comparative impact of video consultation on emergency neurosurgical referrals. Neurosurgery. 2006;59(3):607-613. doi:10.1227/01.NEU.0000228926.13395.F9 16955042

[24] Malhotra S, Chakrabarti S, Shah R, . Telepsychiatry clinical decision support system used by non-psychiatrists in remote areas: validity and reliability of diagnostic module. Indian J Med Res. 2017;146(2):196-204. doi:10.4103/ijmr.IJMR_757_15 29265020 PMC5761029

[25] Ning AY, Cabrera CI, D’Anza B. Telemedicine in otolaryngology: a systematic review of image quality, diagnostic concordance, and patient and provider satisfaction. Ann Otol Rhinol Laryngol. 2021;130(2):195-204. doi:10.1177/0003489420939590 32659100

[26] Hampton JR, Harrison MJ, Mitchell JR, Prichard JS, Seymour C. Relative contributions of history-taking, physical examination, and laboratory investigation to diagnosis and management of medical outpatients. Br Med J. 1975;2(5969):486-489. doi:10.1136/bmj.2.5969.486 1148666 PMC1673456

[27] Peterson MC, Holbrook JH, Von Hales D, Smith NL, Staker LV. Contributions of the history, physical examination, and laboratory investigation in making medical diagnoses. West J Med. 1992;156(2):163-165. doi:10.1097/00006254-199210000-00013 1536065 PMC1003190

