# Error in Supplement 2

**DOI:** 10.1001/jamanetworkopen.2022.39827

**Published:** 2022-10-17

In the Original Investigation titled “Assessment of Clinician Diagnostic Concordance With Video Telemedicine in the Integrated Multispecialty Practice at Mayo Clinic During the Beginning of COVID-19 Pandemic From March to June 2020,”^1^ published September 2, 2022, a contributor’s name was misspelled in Supplement 2. This article has been corrected.^1^
