# Supplementary material for: Assessment of Clinician Diagnostic Concordance With Video Telemedicine in the Integrated Multispecialty Practice at Mayo Clinic During the Beginning of COVID-19 Pandemic From March to June 2020
Source: JAMA Netw Open. 2022 Sep 2;5(9):e2229958. doi: 10.1001/jamanetworkopen.2022.29958 (PMC9440401; doi:10.1001/jamanetworkopen.2022.29958)
Supplement: Supplement 1. — eFigure. Geographic Residence of Patients at Telemedicine Visit eTable 1. Logistic Regression Model Results eTable 2. Concordance Estimates by Clinical Area eTable 3. Concordance Estimates by ICD Chapter eTable 4. Concordance by ICD Code (>10 Cases) [file jamanetwopen-e2229958-s001.pdf]

Supplemental Online Content

Demaerschalk BM, Pines A, Butterfield R, et al; Diagnostic Accuracy of Telemedicine Utilized at Mayo Clinic Alix School of Medicine Study Group Investigators. Assessment of clinician diagnostic concordance with video telemedicine in the integrated multispecialty practice at Mayo Clinic during the beginning of COVID-19 pandemic from March to June 2020. *JAMA Netw Open*. 2022;5(9):e2229958. doi:10.1001/jamanetworkopen.2022.29958

eFigure. Geographic Residence of Patients at Telemedicine Visit
eTable 1. Logistic Regression Model Results
eTable 2. Concordance Estimates by Clinical Area
eTable 3. Concordance Estimates by *ICD* Chapter
eTable 4. Concordance by *ICD* Code (>10 Cases)

This supplemental material has been provided by the authors to give readers additional information about their work.

© 2022 Demaerschalk BM et al. *JAMA Network Open.*

eFigure. Geographic Residence of Patients at Telemedicine Visit

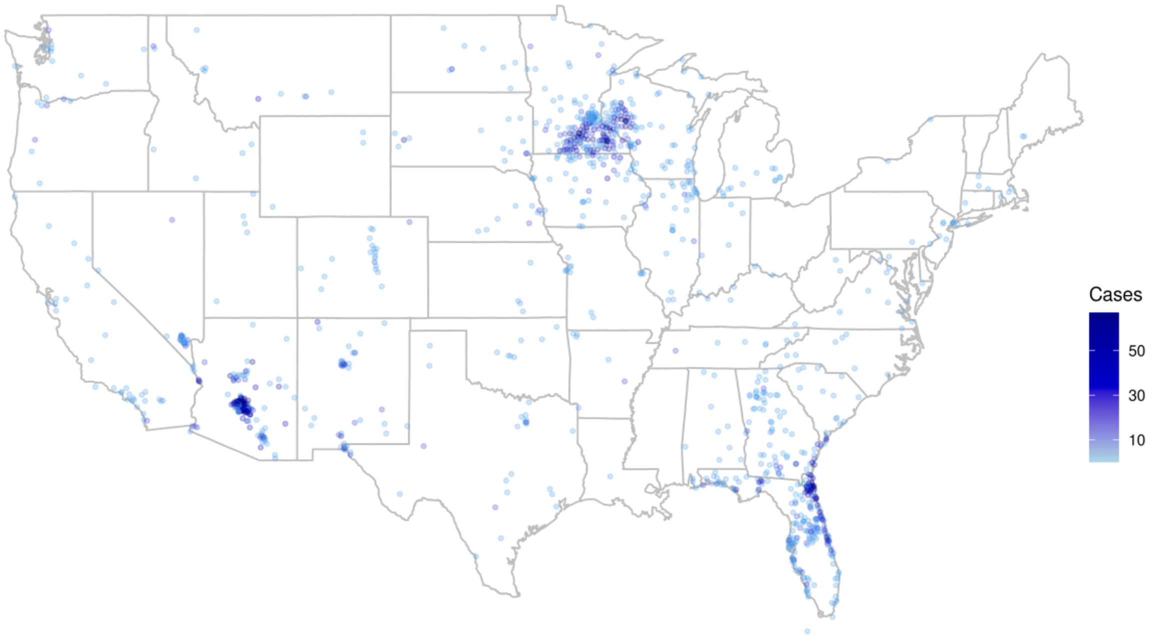

© 2022 Demaerschalk BM et al. *JAMA Network Open.*

**eTable 1. Logistic Regression Model Results**			
**Variable**	**Level**	**Univariate Model**	**Multivariable Model**
		**OR (95% CI), p value**	**OR (95% CI), p value**
Age	Every Ten Unit Increase	0.93 (0.87, 1.00), 0.04	0.91 (0.85, 0.97), 0.007
Gender	Male vs. Female	0.95 (0.74, 1.22), 0.69	
Site	MCHS vs. Arizona	1.38 (0.96, 1.98), 0.09	
	Rochester vs. Arizona	1.24 (0.85, 1.81), 0.26	
	Florida vs. Arizona	1.03 (0.72, 1.46), 0.88	
Clinician Type	Non-Physician vs. Physician	0.99 (0.70, 1.39), 0.95	
Case Type	Surgical vs. Non-Surgical	1.38 (1.02, 1.87), 0.04	1.22 (0.87, 1.71), 0.24
Demographic	Pediatric vs. Adult	0.52 (0.22, 1.21), 0.13	
Specialty	Specialist vs. Primary Care	1.68 (1.27, 2.21), <.001	1.69 (1.24, 2.30), <.001
Duration	Every Ten Unit Increase	1.02 (0.94, 1.10), 0.65	
Clinician Years Certified	Every Ten Unit Increase	0.91 (0.78, 1.05), 0.19	
Prior Telemedicine Experience	Yes vs. No	0.99 (0.68, 1.44), 0.96	
Clinician Years Certified Categories	Certified years 14 - <23 years vs. 0 - <7 years	1.20 (0.79, 1.83), 0.40	
	Certified years 7 - <14 years vs. 0 - <7 years	1.13 (0.75, 1.71), 0.55	
	Certified years >= 23 years vs. 0 - <7 years	0.87 (0.58, 1.29), 0.49	

© 2022 Demaerschalk BM et al. *JAMA Network Open.*

eTable 2. Concordance Estimates by Clinical Area

Level	No. Concordant / No. Cases	Concordance Percent (Wald 95% CI)
Allergy and Immunology	32/33	97.0 (91.1, 100.0)
Breast Clinic	10/11	90.9 (73.9, 100.0)
Cardiovascular Disease	90/105	85.7 (79.0, 92.4)
Cardiovascular Surgery	6/6	100
Child and Adolescent Neurology	6/6	100
Clinical Genomics	2/2	100
Colon and Rectal Surgery	15/16	93.8 (81.9, 100.0)
Community Internal Medicine	76/96	79.2 (71.0, 87.3)
Community Pediatric and Adolescent Medicine	7/10	70
Dermatology	46/58	79.3 (68.9, 89.7)
Endocrinology	10/12	83.3 (62.2, 100.0)
Express or Urgent Care	1/2	50
Family Medicine	245/293	83.6 (79.4, 87.9)
Gastroenterology and Hepatology	192/239	80.3 (75.3, 85.4)
General Internal Medicine	50/63	79.4 (69.4, 89.4)
General Pediatric and Adolescent Medicine	3/7	42.9
General Surgery	18/19	94.7 (84.7, 100.0)
Hematology Oncology	65/65	100.0 (100.0, 100.0)
Hepatobiliary and Pancreas Surgery	4/4	100
Infectious Diseases	5/5	100
Integrative Medicine	3/3	100
Nephrology and Hypertension	5/5	100
Neurological Surgery	80/87	92.0 (86.2, 97.7)
Neurology	114/141	80.9 (74.4, 87.3)
Nutritional Medicine	10/10	100
Obstetrics and Gynecology	102/117	87.2 (81.1, 93.2)
Occupational Medicine	11/12	91.7 (76.0, 100.0)
Oncology and Endocrine Surgery	7/7	100
Ophthalmology	11/12	91.7 (76.0, 100.0)
Orthopedic Surgery	136/149	91.3 (86.7, 95.8)
Otorhinolaryngology	34/44	77.3 (64.9, 89.7)
Pain Medicine	7/7	100
Pediatric Allergy and Immunology	1/1	100
Pediatric Cardiology	7/7	100
Pediatric Gastroenterology	1/1	100
Pediatric Sleep Medicine	3/3	100
Pediatric Urology	1/1	100

© 2022 Demaerschalk BM et al. *JAMA Network Open.*

eTable 2 (Continued). Concordance Estimates by Clinical Area

Level	No. Concordant / No. Cases	Concordance Percent (Wald 95% CI)
Physical Medicine and Rehabilitation	41/48	85.4 (75.4, 95.4)
Plastic Surgery	9/10	90
Preventive Medicine	3/4	75
Psychiatry and Psychology	95/99	96.0 (92.1, 99.8)
Pulmonary Medicine	64/74	86.5 (78.7, 94.3)
Radiation Oncology	67/67	100.0 (100.0, 100.0)
Radiology	1/1	100
Rheumatology	6/9	66.7
Sleep Medicine	24/28	85.7 (72.8, 98.7)
Spine	13/20	65.0 (44.1, 85.9)
Sports Medicine	5/5	100
Thoracic Surgery	15/15	100.0 (100.0, 100.0)
Transplant	105/109	96.3 (92.8, 99.9)
Urology	186/209	89.0 (84.8, 93.2)
Vascular Surgery	7/8	87.5
Women's Health	23/27	85.2 (71.8, 98.6)

© 2022 Demaerschalk BM et al. *JAMA Network Open.*

eTable 3. Concordance Estimates by *ICD* Chapter

Level	No. Concordant / No. Cases	Concordance Percent (Wald 95% CI)
A00-B99: Infectious and Parasitic Diseases	7/8	87.5
C00-D49: Neoplasms	269/278	96.8 (94.7, 98.8)
COVID-19	4/4	100
D50-D89: Blood and Immune Mechanisms	21/24	87.5 (74.3, 100.0)
E00-E89: Endocrine, Nutritional and Metabolic	73/81	90.1 (83.6, 96.6)
F01-F99: Mental, Behavior, and Neurodevelopment	163/172	94.8 (91.4, 98.1)
G00-G99: Nervous System	144/164	87.8 (82.8, 92.8)
H00-H59: Eye and Adnexa	12/14	85.7 (67.4, 100.0)
H60-H95: Ear and Mastoid	11/17	64.7 (42.0, 87.4)
I00-I99: Circulatory System	104/113	92.0 (87.0, 97.0)
J00-J99: Respiratory System	77/90	85.6 (78.3, 92.8)
K00-K95: Digestive System	154/177	87.0 (82.1, 92.0)
L00-L99: Skin and Subcutaneous Tissue	48/63	76.2 (65.7, 86.7)
M00-M99: Musculoskeletal and Connective Tissue	257/300	85.7 (81.7, 89.6)
N00-N99: Genitourinary System	144/169	85.2 (79.9, 90.6)
O00-O9A: Pregnancy, Childbirth and Puerperium	8/8	100
Q00-Q99: Congenital Abnormalities	22/24	91.7 (80.6, 100.0)
R00-R99: Abnormal Clinical Laboratory Findings	303/402	75.4 (71.2, 79.6)
S00-T88: Injury and External Causes	45/49	91.8 (84.2, 99.5)
Z00-Z99: Health Status	208/228	91.2 (87.6, 94.9)

© 2022 Demaerschalk BM et al. *JAMA Network Open.*

eTable 4. Concordance by *ICD* Code (>10 Cases)

ICD Code	ICD Term	No. Concordant / No. Cases	Concordance Percent (Wald 95% CI)
C61	Malignant neoplasm of prostate	56/59	94.9 (89.3, 100.0)
Z00.00	Encounter for general adult medical examination without abnormal findings	21/30	70.0 (53.6, 86.4)
F41.1	Generalized anxiety disorder	28/29	96.6 (89.9, 100.0)
Z01.818	Encounter for other preprocedural examination	23/25	92.0 (81.4, 100.0)
M54.5	Low back pain	17/22	77.3 (59.8, 94.8)
G47.33	Obstructive sleep apnea	16/17	94.1 (82.9, 100.0)
I10	Essential (primary) hypertension	17/17	100.0 (100.0, 100.0)
K21.9	Gastro-esophageal reflux disease without esophagitis	15/17	88.2 (72.9, 100.0)
R97.20	Elevated prostate specific antigen [PSA]	15/17	88.2 (72.9, 100.0)
F33.1	Major depressive disorder, recurrent, moderate	15/15	100.0 (100.0, 100.0)
N40.1	Benign prostatic hyperplasia with lower urinary tract symptoms	12/13	92.3 (77.8, 100.0)
R05	Cough	10/12	83.3 (62.2, 100.0)
C67.9	Malignant neoplasm of bladder, unspecified	11/11	100.0 (100.0, 100.0)

© 2022 Demaerschalk BM et al. *JAMA Network Open.*

eTable 4 (Continued). Concordance by *ICD* Code (6-10 Cases)

ICD Code	ICD Term	No. Concordant / No. Cases	Concordance Percent
F41.9	Anxiety disorder, unspecified	8/10	80
M25.511	Pain in right shoulder	6/10	60
N18.6	End stage renal disease	10/10	100
R13.10	Dysphagia, unspecified	9/10	90
272.4	Other and unspecified hyperlipidemia	9/9	100
C50.412	Malignant neoplasm of upper-outer quadrant of left female breast	9/9	100
F33.0	Major depressive disorder, recurrent, mild	8/9	88.9
G95.9	Disease of spinal cord, unspecified	6/9	66.7
M47.816	Spondylosis without myelopathy or radiculopathy, lumbar region	6/9	66.7
M48.062	Spinal Stenosis Lumbar Region with Neurogenic Claudication	9/9	100
C71.9	Malignant neoplasm of brain, unspecified	8/8	100
E66.01	Morbid (severe) obesity due to excess calories	7/8	87.5
E78.5	Hyperlipidemia, unspecified	8/8	100
K50.90	Crohn's disease, unspecified, without complications	8/8	100
K51.90	Ulcerative colitis, unspecified, without complications	8/8	100
M25.512	Pain in left shoulder	8/8	100
M54.17	Radiculopathy, lumbosacral region	8/8	100
R06.02	Shortness of breath	7/8	87.5
R19.7	Diarrhea, unspecified	8/8	100
R31.9	Hematuria, unspecified	6/8	75
J30.9	Allergic rhinitis, unspecified	7/7	100
L70.0	Acne vulgaris	7/7	100
M25.561	Pain in right knee	7/7	100
N20.0	Calculus of kidney	7/7	100
R56.9	Unspecified convulsions	6/7	85.7
Z00.129	Encounter for routine child health examination without abnormal findings	7/7	100
Z00.5	Encounter for examination of potential donor of organ and tissue	7/7	100
C50.411	Malignant neoplasm of upper-outer quadrant of right female breast	6/6	100
E11.40	Type 2 diabetes mellitus with diabetic neuropathy, unspecified	6/6	100
G43.909	Migraine, unspecified, not intractable, without status migrainosus	6/6	100
G62.9	Polyneuropathy, unspecified	3/6	50
J84.9	Interstitial pulmonary disease, unspecified	6/6	100
M25.50	Pain in unspecified joint	5/6	83.3
M54.12	Radiculopathy, cervical region	6/6	100
M54.2	Cervicalgia	4/6	66.7
N28.89	Other specified disorders of kidney and ureter	5/6	83.3
N39.0	Urinary tract infection, site not specified	4/6	66.7
N80.9	Endometriosis, unspecified	6/6	100
R53.83	Other fatigue	3/6	50

© 2022 Demaerschalk BM et al. *JAMA Network Open.*
