# Supplementary material for: Assessment of Clinician Diagnostic Concordance With Video Telemedicine in the Integrated Multispecialty Practice at Mayo Clinic During the Beginning of COVID-19 Pandemic From March to June 2020
Source: JAMA Netw Open. 2022 Sep 2;5(9):e2229958. doi: 10.1001/jamanetworkopen.2022.29958 (PMC9440401; doi:10.1001/jamanetworkopen.2022.29958)
Supplement: Supplement 2. — Nonauthor Collaborators. Diagnostic Accuracy of Telemedicine Utilized at Mayo Clinic Alix School of Medicine Study Group Investigators [file jamanetwopen-e2229958-s002.pdf]

Supplemental Online Content: Nonauthor Collaborators
*First name, last name, and suffix (if applicable) are required and will appear in PubMed.

*Group Name(s): Diagnostic Accuracy of Telemedicine utilized at Mayo Clinic Alix School of Medicine Study Group Investigators							
***First Name and Middle Initial(s)**	***Last Name**	***Suffix (eg, Jr, III)**	Academic Degrees	Institution	Location (city, state/province, country)	Role or Contribution, eg, chair, principal investigator	Group (if more than 1 Group listed in the byline) and/or Subgroup (eg, Steering Committee)
Stephanie	Anaya		MD	Mayo Clinic Alix School of Medicine	Rochester, Minnesota	Investigator	
Jake	Arbon		MD	University of Utah Health, Psychiatry	Salt Lake City, Utah	Investigator	
Nicholas	Berry		MD	Hackensack Meridian Health Palisades Medical Center, Dermatology	Hackensack, New Jersey	Investigator	
Adip	Bhargav		MD	University of Kansas Medical Center, Neurosurgery	Kansas City, Kansas	Investigator	
Lauren	Breslin		MD	Mayo Clinic Alix School of Medicine	Rochester, Minnesota	Investigator	
Gabriell	Cummings		MD	Mayo Clinic Alix School of Medicine	Rochester, Minnesota	Investigator	
Sam	Ekstein		MD	Mayo Clinic Alix School of Medicine	Rochester, Minnesota	Investigator	
Cecilia	Fung		MD	Mayo Clinic Alix School of Medicine	Rochester, Minnesota	Investigator	
Benjamin	Gorman		MD	Mayo Clinic Alix School of Medicine	Rochester, Minnesota	Investigator	
Maya	Harrington		MD	Mayo Clinic Alix School of Medicine	Rochester, Minnesota	Investigator	
James	Hwang		MD	Mayo Clinic Alix School of Medicine	Rochester, Minnesota	Investigator	
Tyler	Jarvis		MD	Penn State Hershey Medical Center, Plastic Surgery	Hershey, Pennsylvania	Investigator	
Monica	Kirollos		MD	Mayo Clinic Alix School of Medicine	Rochester, Minnesota	Investigator	
Meg	Lang		MD	Mayo Clinic Alix School of Medicine	Rochester, Minnesota	Investigator	
Rachel	Lopdrup		MD	Aurora St. Lukes Medical Center, Family Medicine	Milwaukee, Wisconsin	Investigator	
Sheila	Malekian		MD	University of California Irvine Medical Center, Family Medicine	Irvine, California	Investigator	
Parth Pradip	Patel		MD	University of Pennsylvania, Medicine	Philadelphia, Pennsylvania	Investigator	
Jordan	Pollock		MD	Mayo Clinic Alix School of Medicine	Rochester, Minnesota	Investigator	
Alex	Roth		MD	Mayo Clinic College of Medicine and Science, Psychiatry	Rochester, Minnesota	Investigator	
Hiba	Saifuddin		MD	LSU School of Medicine, Plastic Surgery	New Orleans LA	Investigator	
Serena	Shimshak		MD	Mayo Clinic Alix School of Medicine	Rochester, Minnesota	Investigator	
Daniel	Sykora		MD	Mayo Clinic College of Medicine and Science, Medicine	Rochester, Minnesota	Investigator	
Caitlin	VanLith		MD	Mayo Clinic Alix School of Medicine	Rochester, Minnesota	Investigator	
Samantha	Wilder		MD	Mayo Clinic Alix School of Medicine	Rochester, Minnesota	Investigator	
Samuel	Wu		MD	Mayo Clinic Alix School of Medicine	Rochester, Minnesota	Investigator	
